# Cost-effectiveness analysis of a large-scale crèche intervention to prevent child drowning in rural Bangladesh

**DOI:** 10.1186/s40621-021-00351-9

**Published:** 2021-10-29

**Authors:** Y. Natalia Alfonso, Adnan A. Hyder, Olakunle Alonge, Shumona Sharmin Salam, Kamran Baset, Aminur Rahman, Dewan Md Emdadul Hoque, Md Irteja Islam, Fazlur Rahman, Shams El-Arifeen, David Bishai

**Affiliations:** 1 Department of International Health, Johns Hopkins Bloomberg School of Public Health, Baltimore, MD USA; 2 Milken Institute School of Public Health, George Washington University, Washington, DC USA; 3 Department of Oncology and Metabolism, University of Sheffield, Sheffield, UK; 4 Center for Injury Prevention and Research, Bangladesh (CIPRB), Dhaka, Bangladesh; 5 Maternal and Child Health Division, International Center for Diarrhoeal Diseases Research, Bangladesh, Dhaka, Bangladesh; 6 Department of Population, Family and Reproductive Health, Johns Hopkins Bloomberg School of Public Health, Baltimore, MD USA

**Keywords:** Drowning, Children, Mortality, Injury, Child health, Crèche, Prevention, Cost-effectiveness, Bangladesh

## Abstract

**Background:**

Drowning is the leading cause of death among children 12–59 months old in rural Bangladesh. This study evaluated the cost-effectiveness of a large-scale crèche (daycare) intervention in preventing child drowning.

**Methods:**

The cost of the crèches intervention was evaluated using an ingredients-based approach and monthly expenditure data collected prospectively throughout the study period from two agencies implementing the intervention in different study areas. The estimate of the effectiveness of the crèches intervention was based on a previous study. The study evaluated the cost-effectiveness from both a program and societal perspective.

**Results:**

From the program perspective the annual operating cost of a crèche was $416.35 (95% CI: $221 to $576), the annual cost per child was $16 (95% CI: $8 to $23), and the incremental-cost-effectiveness ratio (ICER) per life saved with the crèches was $17,008 (95% CI: $8817 to $24,619). From the societal perspective (including parents time valued) the ICER per life saved was − $166,833 (95% CI: − $197,421 to − $141,341)—meaning crèches generated net economic benefits per child enrolled. Based on the ICER per disability-adjusted-life years averted from the societal perspective (excluding parents time), $1978, the crèche intervention was cost-effective even when the societal economic benefits were ignored.

**Conclusions:**

Based on the evidence, the crèche intervention has great potential for generating net societal economic gains by reducing child drowning at a program cost that is reasonable.

**Supplementary Information:**

The online version contains supplementary material available at 10.1186/s40621-021-00351-9.

## Introduction

Drowning is one of the top five causes of death for children aged 1–14 in 48 out of 85 countries with data on drowning deaths (World Health Organization 2014). The global burden of fatal drownings disproportionally affects low-and-middle-income countries (LMICs), with approximately 7 to 8 drowning deaths per 100,000 people in sub-Saharan Africa and Southeast Asia compared to 2 to 3 in high-income countries (World Health Organization 2014). Bangladesh, has one of the highest drowning rates among children aged 12–59 months (equivalent to 58% of deaths among children of this age) (National Institute of Population Research and Training (NIPORT) and ICF 2020). Despite Bangladesh’s success in reducing under-five all-cause mortality by two thirds during the past decade (Chowdhury et al. 2013), drowning death rates have remained generally the same (Rahman et al. 2019). Child drowning affects households across all regions of Bangladesh, particularly in rural areas, and is associated with household behaviors and various contextual factors (Rahman et al. 2017). Tackling these factors would require scale up of cost-effective childhood drowning prevention programs at the national level (Rahman et al. 2017). More evidence on the cost-effectiveness of drowning prevention interventions is needed to get political support to allocate scare resources to these programs across the country.

Rural Bangladeshi children are at high risk of drowning because homes are generally located in areas surrounded by natural bodies of water (Hyder et al. 2008). In these settings, child caregivers are less able to mitigate the drowning risk posed by the large water bodies (Hyder et al. 2008). Most of these drownings occur in ponds and ditches between 9am and 1 pm when caregivers are busiest with household chores (Rahman et al. 2009). Interventions tailored to reduce drowning in LMICs focus on reducing children’s unsupervised access to water bodies, included door barriers, playpens, swimming lessons and crèches (i.e., child care centers) (Hyder et al. 2014a). In 2012, a study conducted in rural Bangladesh evaluated the cost-effectiveness of a drowning prevention package, including swimming lessons and crèches. The study found that the package of interventions reduced the relative risk of drowning in under-five age children by 89% (Rahman et al. 2012). However, the study did not disentangle the effects of the swimming lessons and crèches interventions.

The high burden of drowning in Bangladesh and other LMIC countries requires urgent action from community leaders and policy makers to implement or scale up effective and efficient drowning prevention strategies. As noted by Mayor Michael Bloomberg, the government of Bangladesh is considering a policy of expanding access to daycare services as an evidence-based strategy to prevent drowning (Bloomberg 2021). More evidence on the cost-effectiveness of the strategies proposed and tailored to the specific needs of each setting are needed to identify and advocate for the interventions that can best mitigate the risk of drowning (World Health Organization 2014; Hyder et al. 2008; Callaghan et al. 2010; Royal Life Saving, Alliance for Safe Children 2021). This evidence would help guide policy makers on what interventions will save more lives efficiently and effectively.

Assessment of the costs and cost-effectiveness of community interventions requires scientific evidence on program impact and comprehensive data on all the resources needed to implement and scale interventions. This study took advantage of the implementation of a large-scale crèche intervention in rural Bangladesh to incorporate prospectively a cost-effectiveness analysis (CEA) into the evaluation of the intervention (Bloomberg 2021; Callaghan et al. 2010). The objective of this study was to evaluate the cost-effectiveness of the crèche intervention for reducing drowning deaths among children aged 1 to 4 years in rural Bangladesh. This paper answered the question on whether the crèche intervention is cost-effective compared to the status-quo from both the program and societal perspectives.

## Methods

### The intervention

The crèche intervention was a community-based program designed to reduce child drowning in rural communities among children aged 9 to 47 months old. The crèche program was a tuition-free daycare service run by two volunteer females (a crèche caregiver and an assistant). The word crèche is used throughout this study, rather than daycare or childcare, because this intervention is widely known as crèches in Bangladesh. Each crèche supervised an average of 26 children during peak drowning hours, 9am to 1 pm, six days a week, and provided learning activities on language, numbers, drawing, dancing, health, and injury prevention, among other activities for development of cognitive and motor skills (Hyder et al. 2014b; Alonge et al. 2020). Crèche caregivers (i.e., referred to as crèche “Ma”) were trained for five days and assistants were trained for one day. Each crèche was also supervised by a crèche supervisor 3–5 times each month. Local village and union injury prevention committee (VIPCs and UIPCs) members met at least once a month throughout the study period to plan sensitization of the community about injury prevention and engagement of parents in the crèches program. The committee members were elders known and respected by the local community and worked closely with the village parents to nominate potential crèche Mas, assistants, and crèche sites. VIPCs also helped the program staff to monitor crèche operations, support crèche Mas’ and parents’ needs, and getting their feedback.

Crèche Mas voluntarily offered space in their homes to run a crèche center. Program supervisors screened each site and only those that met criteria for safety, cleanliness, and child appropriateness were selected. The program provided initial investments for minor repairs (e.g., doors, fans, lighting improvements, floors etc.) to ensure strict safety requirements. Each crèche site space was one room with secured doors and windows, adequate light, and ventilation, and equipped with carpeted floors, age-appropriate toys, and educational supplies. Maintenance of space and supplies (e.g., soap, toys, food containers, recurrent repairs, etc.) were also provided on an annual basis. Details about the crèche program are provided elsewhere (Hyder et al. 2014b; Alonge et al. 2020).

### Study population and data

This study obtained program costs and drowning data from the Saving of Lives from Drowning (SoLiD) cohort study which implemented the crèche intervention and an injury surveillance system in two rural areas in Bangladesh with the collaboration of the Centre for Injury Prevention and Research, Bangladesh (CIPRB) and International Centre for Diarrhoeal Disease Research, Bangladesh (ICDDRB). Area 1 included three sub-districts: Sherpur, Manohardi, and Raiganj. Area 2 included four sub-districts: Matlab North, Matlab South, Daudkandi and Chandpur. These sub-districts were purposely selected for their prior high drowning rates and the local partners’ experience working with communities in these areas. Crèche centers were established in every village creating capacity for all children ages 9–47 months old residing in the study areas. All parents in study sites were encouraged to enroll their children of this age group. A total of 3205 functional crèches were established in 451 villages with about 1.2 million people (Alonge et al. 2020).

The primary health outcome for the CEA was drowning deaths averted among children 12–47 months old. Drowning and crèche program participation data were collected from quarterly injury surveillance data and a baseline survey administered to all households in the study areas. Additional details about the study population, questionnaire design and data collection procedure are provided elsewhere (Hyder et al. 2014b; Alonge et al. 2020). Enrollment started in June 2013 and continued through 2015. A one-year time horizon was used to evaluate program effects and a 3-year horizon was used to evaluate program costs. This time horizon captured changes in the annual cost over time due to gains in efficiency as program staff became more experienced in running the program.

### Effectiveness

The effectiveness of the crèche intervention in reducing the annual cumulative death rate from drowning was obtained from Alonge et al. 2020. That effectiveness study used a pre-and-post intent-to-treat study design and the analysis adjusted for major sources of bias and confounding, particularly selection bias and secular trends. The analysis controlled for self-selection bias by adjusting for household fixed effects and an analysis of historical trends showed that other local interventions were unlikely to have impacted the results. To control for household fixed effects the study compared the cumulative incidence of drowning between the eligible children during the treatment period (i.e. treated sample) and historical data on ineligible siblings and pre-treatment data on eligible children during the 12 months prior to the study baseline (i.e. untreated sample) (Alonge et al. 2020). This study produced estimates of the change in annual cumulative drowning death rate by age group. Furthermore, historical drowning rates between the years 1998 to 2012 from the study areas showed that while all-cause child mortality declined over a 14-year period prior to the crèches study, drowning-specific mortality remained largely unchanged (Alonge et al. 2020, 2017a). Thus, supporting the claim that the observed effect in this study area were likely due to the crèche intervention and not a secular trend. The study found that risk ratios (RR) of drowning deaths with the intervention were 0.40 (95% CI: 0.28–0.57) overall. However, age specific risk ratios were stronger for children over 3 and weaker for children between age 1 and 2. We applied age-specific  RR to a synthetic cohort of 100,000 children aged 1–4 to estimate deaths averted. We used the 95% confidence-range around the RR’s from the effectiveness study to produce an uncertainty range for deaths averted and varied these values in sensitivity analyses (SA) (Alonge et al. 2020). Details about the SA of the effectiveness estimate are provided below.

We converted deaths averted to years-of-life-lost (YLLs) to provide a conservative approximation of disability-adjusted-life-years (DALYs) averted. In the Bangladeshi context, emergency care is limited, and children pulled out of the water after being submersed either survive with no disability or die within 24 hours. As a first approximation, years lived with disability after a submersion in this setting are zero and so YLLs are nearly equivalent to DALYs. YLLs were estimated using the World Health Organization (WHO)’s DALY calculation template (World Health Organization 2003, 2020). Parameters in the calculation included the study population’s average age of drowning, 2.3 years old, life expectancy at birth, 72.2 (United Nations Population Division 2020) years, and a standard three percent discounted rate for future years of life lost.

### Costs

The analysis of costs was conducted using an ingredients-based approach. With this methodology all program expenditures were recorded by category throughout the study period. Costs are evaluated from both a program and a societal perspective following the recommendations set by the Second Panel on Cost-Effectiveness in Health and Medicine (Sanders et al. 2016). Program expenditures were categorized into the following major groups or “program inputs”: start-up costs, equipment and trainings, rent, wages, community engagement/sensitization, transportation, field overhead, and administration operations. Both program costs and the count of children enrolled (i.e., exposed to the program) were collected monthly in real time from program accountants and revised by program managers. For a detailed description of each program input see the Additional file 1 exhibit A. A total of 54-month observations of data were collected including 31 months from Area 1 (from June 2013 to December 2015) and 23 months from Area 2 (from February 2014 to December 2015).

Informant interviews were conducted with local program supervisors throughout the study period to capture the percent effort of staff and program inputs attributed to the crèche versus other research or program activities. The cost analysis excluded research and non-crèche program costs. Cost data was inflation-adjusted to 2015 BDT and currency converted to US dollars (USD) in 2015. Shared costs were apportioned to the crèche program based on the crèches’ shared percent effort. Fixed costs (i.e., start-up costs, trainings, and equipment) were discounted with a standard three percent rate and annualized using program experts’ advice on inputs’ lifetime. Program cost data from each month was compared against the number of children enrolled the same month to produced monthly total and average cost estimates and trends. The monthly cost trend was then used to assess how expenditures varied over the study period as the program reached maximum capacity and became more experienced, as well as to differentiate between the cost during a start-up year (i.e., when initial investments are spent) versus an annual operational cost. These estimates were used to model a 10-year projection of the program cost in which investments in fixed costs were annualized and investments in variable costs were the cost of a program running at mid-to-full capacity. Estimates from the 10-year expenditure model were then averaged over the ten years to estimate the annual total cost and average cost per child.

Societal costs added the opportunity cost of the crèche Ma’s and assistant’s time, VIPC and UIPC members’ time, and economic savings from a parent’s improved productivity. Regarding the economic savings, currently the total fertility rate in Bangladesh is 2.06 and birth intervals are 47 months, thus most households with any children old enough to attend a crèche would be unlikely to have a remaining younger child ineligible for school or crèche (Khan et al. 2016). This implies that a crèche would free up four hours a day, 6 days a week. Guidance from the Second Panel on Cost-Effectiveness says that, “…economic theory implies clearly that the value people place on an hour of their leisure time can be inferred from their hourly wage…”. We recognize that rural Bangladeshi villages offer negligible opportunities for women to engage in wage-labor, however the activities that women will do with the extra 24 h per week freed up from child-care responsibilities will have value to them and their households. Exactly how the mothers of enrolled children will spend their freed-up time is unknown. According to economic theory the value of an hour of leisure time is equal to the wage rate when a labor market is at equilibrium. So as a placeholder approach the value of freed-up mothers’ time was estimated based on  the Bangladeshi minimum wage. Assuming a work week was 6 days (48-h), and a women with a child in crèches was freed up 4 h per day (i.e., half the work-week), then her opportunity cost of time was 50% of the minimum wage of 8000 BDT (equivalent to $78.5 in 2015) (Butler 2020). Parents’ improved productivity assumed one parent per child participant benefited producing economic benefits. Similarly, for the subset of women with children attending a crèche less than 6 days per week, we valued their time by a fraction of the opportunity cost of time according to the utilization rates obtained from the study records (Alonge et al. 2020). Thus, the cost of parents’ time was a function of both the minimum wage value and monthly attendance rate. Details about the SA of parents’ time are provided below.

Similarly, the crèche worker’s honorarium of $27.38 per month was far lower than the minimum wage. Although crèche staff participated mostly out of a sense of voluntarism, guidelines mandate that the value of their time appeal to market rates (Neumann et al. 2016). Because crèche workers worked only half days and to avoid an under-estimate of program costs from the societal perspective, we proxy opportunity cost of the crèche Ma and assistant as half (for part-time) of the minimum wage, instead of $27.38 per month and per crèche.

The cost of VIPC and UIPC members’ time was estimated as the product of person-months per crèche and the cost per person-month. The number of person-months per crèche was the product of the number of members per meeting and 12 months. The cost per person-month was estimated as  the number of meetings/month x hours/meeting x minimum wage/hour. The wage/hour was the hourly rate equivalent of the minimum wage described previously. We also assumed the fixed values of 3-h per meeting and at least 7 members per meeting based on experts’ opinion. Thus, the cost of VIPC and UIPC members’ time varied as function of the wage rate and the number of meetings per month per crèche. Given that the amount of effort put in by VIPC and UIPC members in community sensitization activities could have contributed to the usage of the crèches, we varied these two parameters in SA.

This cost analysis excluded the cost of the healthcare services that would have been provided to drowning survivors. This cost input was excluded because the number of injured near-drowning survivors in rural Bangladesh is very small to negligible. However, in other settings, particularly higher income countries, where urgent healthcare services for drowning victims may be more accessible in rural areas, it would be important to include these inputs in the estimation of societal costs. Particularly, including the cost of time and resources of both the village responders and healthcare services. This issue is further described in the limitations section of the discussion. Societal costs were estimated per crèche and child enrolled. For details about assumptions see the Additional file 1.

### Cost-effectiveness analysis

The incremental-cost effectiveness ratio (ICER) was calculated by dividing the incremental cost between the crèche intervention and the status-quo by the incremental effect size (i.e., deaths averted with the intervention) for a hypothetical population of 100,000 children aged 12 to 47 months old (Drummond et al. 2015). ICERs were estimated from both the program and societal perspectives.

### Sensitivity analysis

Given that both the effectiveness and cost parameters used in cost-effectiveness analyses have inherent uncertainty, we used a Monte Carlo simulation to produce confidence intervals around the ICER and the total and average program cost estimates (Buckland 1984). The simulation included 100,000 iterations of the cost-effectiveness analysis model. First, we used statistical analysis software (@Risk 2019) to fit the distribution of observations of data of each input to the best probability density function (e.g., Normal, Gaussian, Poisson, Log-normal, etc.). Then, each iteration used a different set of random values from each probability function. The simulation varied the model inputs that significantly affected the ICER results (i.e., each category of program costs, the effectiveness, the minimum wage, the number of VIPC and UIPC meetings per month, and the number of children enrolled per crèche). Other values, such as VIPC/UIPC meeting hours and members per meeting, were fixed in the simulation.

The data used to find the best function fit included 54-monthly observations of expenditures for each cost input and the crèche attendance. Other inputs, except wages, included 31-monthly observations of data. For wage data, given that the range of these values for different localities in Bangladesh was not available, we used 10-yearly observations of gross-domestic product per capita in Bangladesh between 2010 and 2019 (inflation adjusted to 2015 USD) and assumed that the distribution function of this data resembled the variation of rural minimum wages (i.e., cost of living) in rural Bangladesh. The mean of the probability functions was centered at the mean estimated by the cost analysis so that  the simulation matched with the costing model. Each probability function was also truncated at the minimum and maximum values observed in the data.

The interventions’ effectiveness varied in the simulation in the same way as the cost inputs, but in this case, we assumed that the effect size estimate had a lognormal distribution. The mean of the distribution was the mean deaths averted and the standard deviation was derived from the 95% CI values. The standard deviation was estimated as (upper bound − lower bound)/(2 * 1.96), which is the same as the standard error for data derived from rates. See the Additional file 1 exhibit B1-3 for details about assumptions for the sensitivity analysis. The Monte Carlo simulation and identification of the distributional function of each input were obtained using the @Risk software version 8.0 (@Risk 2019).

Additionally, univariate sensitivity analyses were done by varying program cost and effect inputs by one standard deviation to assess which input had the greatest impact on the ICER (Drummond et al. 2015). Program costs and lives saved were also changed linearly to assess how changes affected the ICER. All analyses and reporting of results were conducted in accordance with the guidelines provided by the Consolidated Health Economic Evaluation Reporting Standards (CHEERS) checklist. Out of the 24 categories, we met all applicable standards, see the Additional file 1 for the checklist (Husereau et al. 2013).

## Results

### Effectiveness

At baseline, the mortality rate from drowning ranged between 125.45 (95% CI 87–181) to 100.55 (95% CI 67–151) per 100,000 children depending on the child’s age, see Table 1. A total of 58% of the children ever attended the crèches (of which 63% attended five or more days per week, 27% attended 2 to 4 days per week, and 10% attended less than 2 days). The crèches intervention reduced the mortality rate from drowning to rates between 34.00 (95% CI 13–90) and 4.00 (95% CI 0.2–60) per 100,000 children depending on the child’s age, see Table 1 for mortality rates by age group (Alonge et al. 2020). Based on these estimates, in a hypothetical population of 100,000 children aged 12 to 47 months old, 111.00 would fatally drown *without* the crèches intervention and 15.60 would fatally drown with the intervention implying an estimate of 95.41 deaths averted per 100,000 children with the intervention. The total number of DALYs per fatal under-five drowning was 29.23, for a total of 2788.59 DALYs averted per every 100,000 children.

**Table 1:** Population parameters

Age groups	Drowning cumulative incidence per 100,000 children per age category and year^†^			Model of hypothetical population of a total of 100,000 children exposed to crèches per year^‡^				
	Cumulative incidence	95% CI		Hypothetical population*	Total deaths	95% CI		SD
*Status-quo*								
12–23 months old	125.45	87.19	180.47	32,898	41.27	28.68	59.37	7.83
24–35 months old	107.10	72.93	157.26	34,548	37.00	25.20	54.33	7.43
36–47 months old	100.55	66.82	151.26	32,554	32.73	21.75	49.24	7.01
Total	–	–	–	100,000	111.00	75.63	162.94	22.27
*Crèches*								
12–23 months old	34.00	13.00	90.00	32,898	11.19	4.28	29.61	6.46
24–35 months old	9.00	2.00	36.00	34,548	3.11	0.69	12.44	3.00
36–47 months old	4.00	0.20	60.00	32,554	1.30	0.07	19.53	4.97
Total	–	–	–	100,000	15.60	5.03	61.58	14.42

Change				Hypothetical population*	Lives saved	95% CI		SD
12–23 months old				32,898	30.09	24.41	29.76	1.37
24–35 months old				34,548	33.89	24.50	41.89	4.44
36–47 months old				32,554	31.43	21.69	29.71	2.05
Total				100,000	95.41	70.60	101.36	7.85

^†^Data from Alonge et al. (2020)

^‡^Author's calculations

*Out of 100,000 children in Bangladesh 32.90% were 12 to 23 months old, 34.55% were 24 to 35 months old, and 32.55% were 36 to 47 months old, source is Alonge et al. (2020)

### Cost

**Fig. 1:**
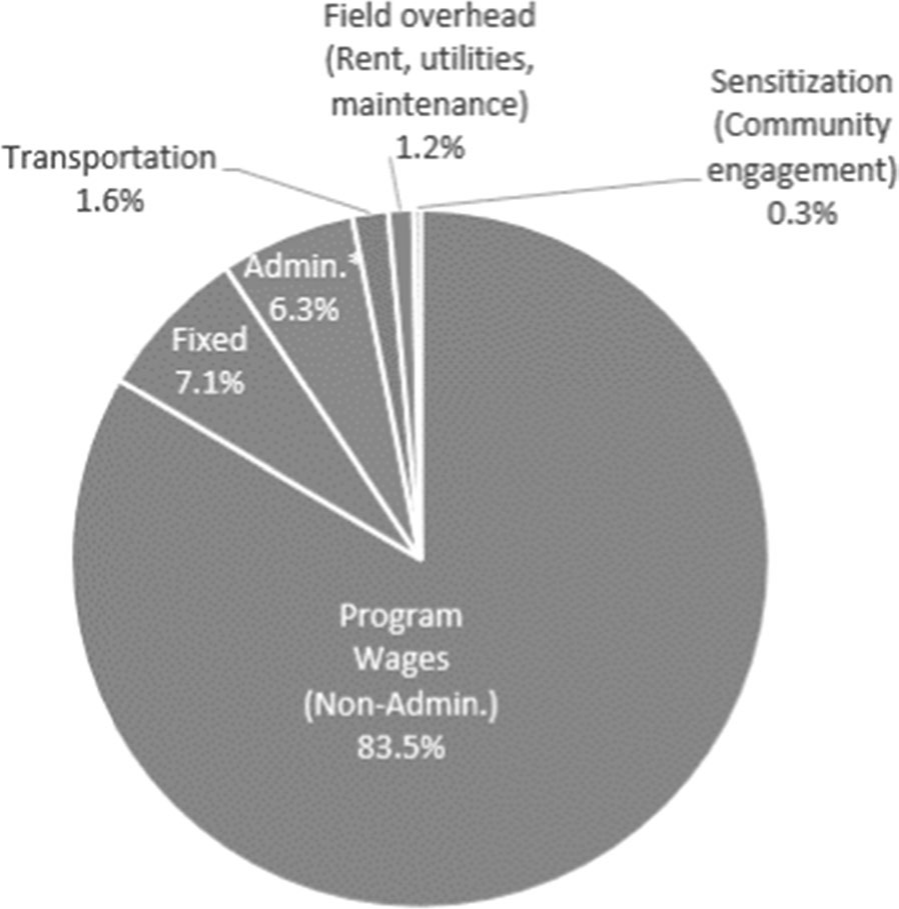
Proportional distribution of crèche program costs

Figure 1 shows the percent distribution of the crèche program costs by major input category. Non-administrative wages made the majority, 83%, of the total program cost. Based on the 10-year projection model, the crèche’s total annual cost for setting-up and running 1554 crèche centers (i.e., the average number of functional crèches per study area) serving an average of 26 children per crèche was $647,074. See the Additional file 1 exhibit C and D for details about annual estimates used in the 10-year cost projection. A total of 40,378 children were enrolled in 1554 crèche centers. The annual average cost per crèche was $416.35 (95% CI $221-$576) and the average cost per child was $16.03 (95% CI $9-$23), respectively. The equivalent monthly average cost per crèche and child was $34.70 and $1.34, respectively. Table 2 provides a summary of program output and cost parameters. For details about cost estimates and time trend by study area see Additional file 1 exhibits E and F.

**Table 2:** Program output and cost parameters

Parameters	Model of annualized costs*	Monthly data from two study areas (54-month observations)				Source
Program Output	Annual total	Monthly total	SD	Min	Max	
Functional crèches	1554	1554	585	9	1600	Program data
Total caseload (children enrolled/exposed)	40,378	40,378	15,572	166	47,992	ibid
Children per crèche	26	26	6	18	30	Authors' calculations
**Program Cost (in 2015 US$)**						
*Fixed cost (FC)* †						
Start-up	$534	$44	**-**	**-**	**-**	Program data and authors' calculations using 54-observations of monthly expenditures, including data from both study areas in Bangladesh
Equipment (administration)	$203	$17	$135	$0	$964	
Trainings	$3356	$280	$862	$0	$4820	
Crèche maintenance (stationery supplies and repairs)	$41,803	$3484	$12,032	$56	$74,719	
FC sub-total	$45,896	$3825	-	$168	$75,191	
*Variable cost (VC)*						
Wages and stipends (field staff)	$540,612	$45,051	$16,329	$687	$50,825	
Community engagement (sensitization, UPICs, VPICs)	$1959	$163	$149	$0	$1013	
Transportation	$10,662	$889	$490	$45	$2940	
Field overhead (rent, utilities, maintenance)	$7497	$625	$313	$110	$1020	
Administration (wages, rent, communication, maintenance)	$40,448	$3371	$1241	$2348	$8406	
VC sub-total	$601,179	$50,098	–	$4178	$55,362	
*Total cost*	$647,074	$53,923	–	$6728	$92,004	
**Average cost**						
Per crèche	$416.35	$34.70	$171.52	$32.54	$847.37	Authors' calculations
Per child	$16.03	$1.34	$7.88	$1.08	$40.53	Ibid

*See the Additional file 1 for details about the model of annualized costs. †Useful life of fixed costs for startup, equipment, trainings, and crèche maintenance were assumed to be 10, 7.5, 5 and 3 years respectively. Parameters with black bold text indicate major parameter groups: program output, program cost, average cost. Parameters in italic text indicate type of cost group: fixed cost, variable cost, total cost

Table 3 provides a summary of the societal cost estimates and assumptions. The number of volunteer hours per year required from crèche workers and VIPC/UIPC members was 2304 and 139 h, respectively. The average parent freed up time per crèche was 3393 h. The annual cost of the crèche Ma and assistant was $36.25 per child enrolled, and of the VIPC and UIPC members’ time was $2.18 per child enrolled. The opportunity cost of caregivers freed up time was $213.48 per child enrolled. With the value of parents freed up time included, the crèche program generates a total of $159.02 in economic value per child enrolled from the societal perspective. If one takes the view that parents freed up time is of no value to society then the program’s societal cost is ($36.25 + $2.18 + $16.03) $54.46 per child per year.

**Table 3:** Societal cost

Parameters (in 2015 US$)	Annual volunteer-hours per crèche	Annual cost per crèche	Annual cost per child
Opportunity cost of time*			
Crèche workers' time (mother and assistant)^†^	2304.00	$941.76	$36.25
VIPC and UIPC members' time	138.72	$56.70	$2.18
Parents' time^‡^	3392.26	− $5546.35	− $213.48
**Program cost**			
Estimates from Table 2	–	$416.35	$16.03
**Total cost (savings)**			
Opportunity cost of time + program cost		− $4131.54	− $159.02

Negative values indicate savings. VIPC and UIPC are Village and Union Injury Prevention Committees. See the Additional file 1 for details about data assumptions and calculations. Assumptions are varied in sensitivity analysis

*The opportunity cost of time is the minimum wage, 8000 BDT per month in the year 2018 based on Butler (2019), equivalent to US$78.48 in 2015

^†^A crèche operates for 4 h per day, 6 days per week, which is part-time, thus the wage per crèche worker is 50% of the minimum wage

^‡^Assumes savings for only one parent per child and the estimate is a function of the minimum wage and crèche attendance level, see the Additional file 1 for details. Parameters with black bold text indicate major parameter groups: opportunity cost, program cost, total societal cost

### Cost-effectiveness

Scaled up to a hypothetical population of 100,000 children aged 12–47 months, at $16.03 per child exposed, the total program cost was $1,602,556 and it would save 95.41 lives per year. Comparing the crèches program to the status-quo, the incremental cost per life saved (ICER) was $16,797 (95% CI $8829–$24,461) from the program perspective, see Table 4. The societal perspective results depend on whether the economic value of parents freed up time is included. With the value of time included the program generated $166,679 (95% CI $196,649–$141,569) in economic benefit, as well as saving 95.41 lives, which averts 2788.59 DALYs. However, if parents freed up time is not valued, the ICER was $57,078 (95% CI $44,346–$72,602) per life saved. Expressed in $ per DALY averted, the ICER was $575 from a program perspective, or − $5703 (i.e., generated savings) from a societal perspective if parents’ time is valued. If parents’ time is not valued the ICER from a societal perspective was $2953 per DALY averted.

**Table 4:** Incremental cost-effectiveness ratio

Cost-effectiveness Analysis (in 2015 US$) Comparison between the intervention and status-quo by program perspective	Incremental cost per year*	Incremental effect per year*	Incremental cost-effectiveness ratio, ICER (95% CI)	
ICER per live saved		Lives saved	Per live saved	
*Program perspective*				
Crèche Intervention	$1602,556	95.41	$16,797	($8829 to $24,461)
*Societal perspective*				
Crèche intervention (Parents' time valued)	− $15,902,459	95.41	− $166,679	(− $196,649 to − $141,569)
Crèche intervention (Parents' time not valued)	$5,445,670	95.41	$57,078	($44,346 to $72,602)

ICER per DALY averted		DALYs averted	Per DALY averted	
*Program perspective*				
Crèche Intervention	$1,602,556	2788.59	$575	($302 to $837)
*Societal perspective*				
Crèche intervention (Parents' time valued)	–$15,902,459	2788.59	− $5703	(− $6728 to − $4844)
Crèche intervention (Parents' time not valued)	$5,445,670	2788.59	$1953	($1517 to $2484)

Negative values indicate savings

*Estimates for a hypothetical population of 100,000 children aged 12 to 47 months old

### Sensitivity analysis

In order to determine if an intervention is cost-effective, one needs a country specific acceptability threshold that had been determined in a way to reflect local values. Whereas other countries have developed official thresholds, no such threshold exists for Bangladesh. Basing a threshold on gross domestic product (GDP) per capita is no substitute for using a country’s stated acceptability threshold. However, if one were to refer to Bangladesh’s GDP per capita of $1248.48 per year (in 2015 USD) and a value of 3 × GDP of $3745.44 one could conduct sensitivity analysis to see what would be required for the program to exceed the 3 X GDP threshold. We find that from the societal perspective (parents time not valued) the cost per child enrolled would have to increase by more than 90%, from $54.46 to $103.47 (Additional file 1 exhibit G1a). Similarly, the program effect size would have to decrease by 52%, from 95.41 to 45.41 lives saved, for the program to fall from the category of very cost-effective to just cost-effective, or by 84%, from 95.41 to 15.41, to become not cost-effective. See Additional file 1 exhibit G2 for figures showing the linear relationship between the ICER and the program unit cost and lives saved (or DALYs averted).

**Fig. 2:**
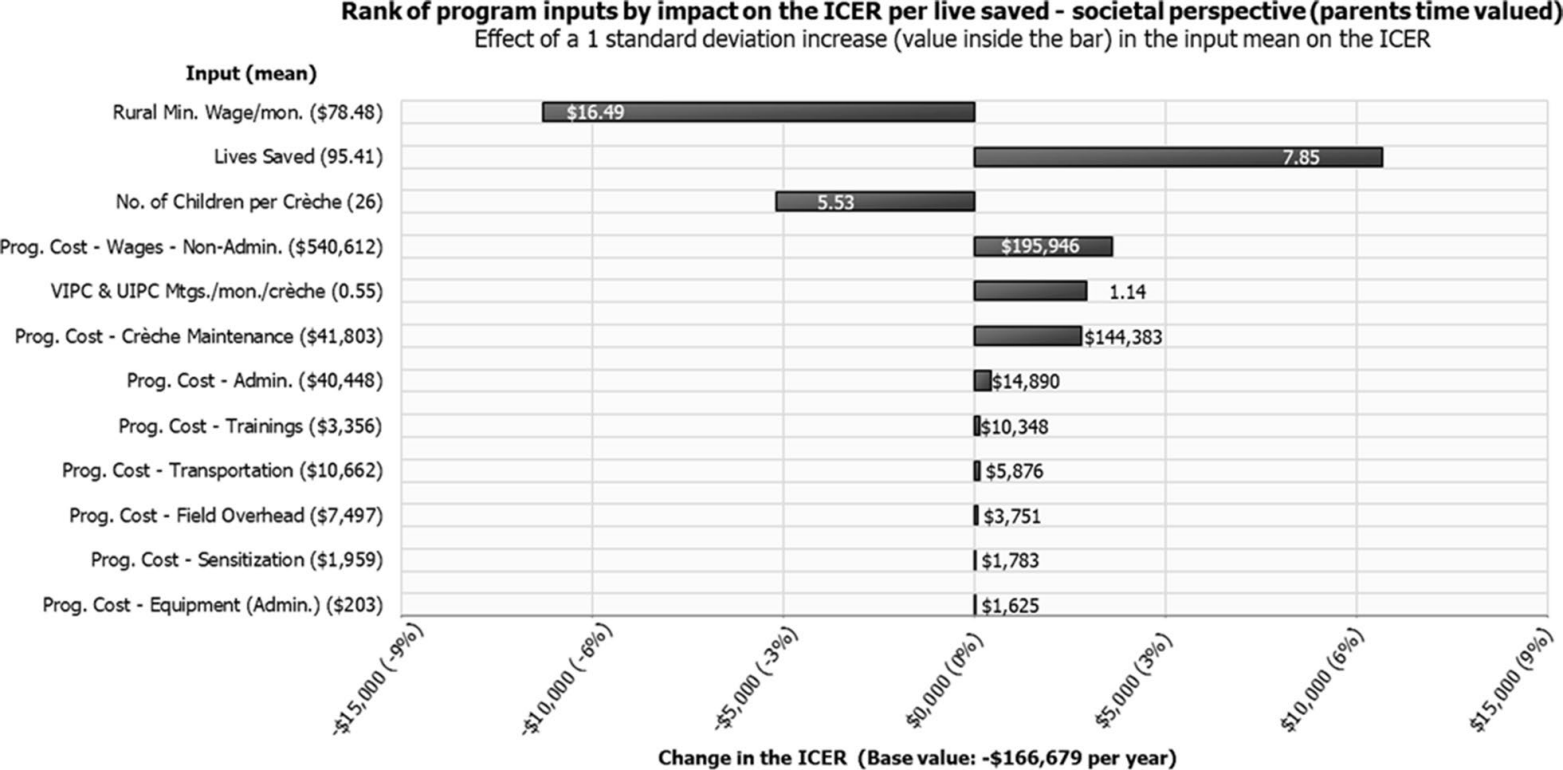
Sensitivity analysis of the effect of changes to program parameters on the ICER. *Note*: An example of how to interpret this graph is, when the monthly minimum wage rate increased by $16.49 (1 standard deviation), the ICER per live saved cost savings increased approximately by 7%, or when the number of lives saved increased by 7.85 lives (1 standard deviation), the ICER per live saved cost savings decreased approximately by 6%

Figure 2 shows program inputs ranked by effect on the ICER mean. Each input is varied by 1 SD (the value shown on each bar), and the x-axis shows the percent change in the ICER (per live saved). From a societal perspective, among the inputs, the minimum wage (which affects the cost of parents’, VIPC/UIPC members’, and crèche caregivers’ time) was the most important factors affecting the ICER. This was followed by lives saved, the number of children per crèche, and program costs. Among the program cost inputs, the non-administrative program wages, the number of VIPC/UIPC meetings per crèche, and maintenance cost were the most important factors affecting the ICER.

**Fig. 3:**
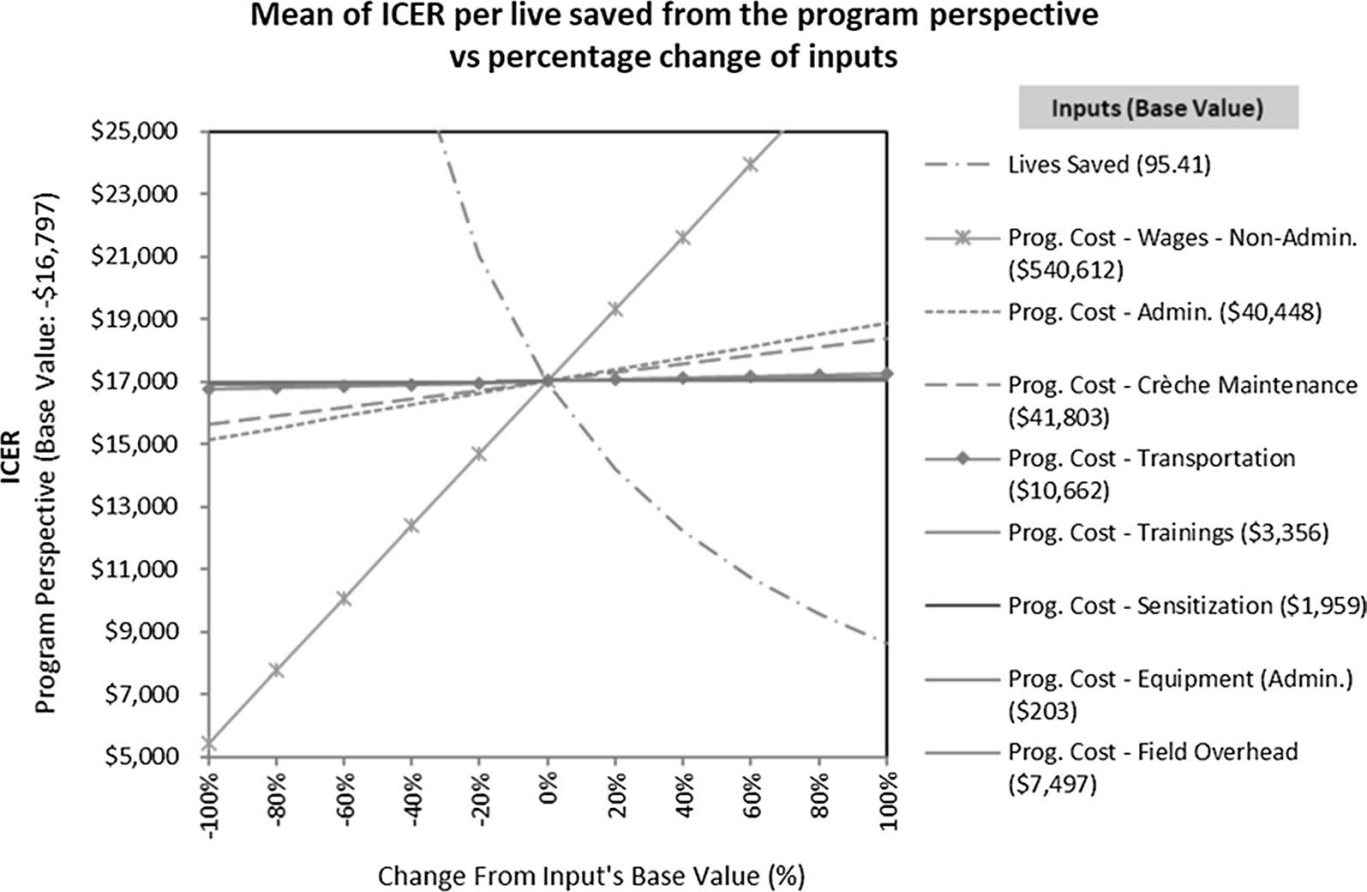
Effect of proportional changes to the program parameters on the ICER

Figure 3 shows how proportional changes in increments of 20% to each program parameter changed the ICER (per live saved). From a program perspective, decreasing the number of lives saved by 20% would increase the ICER from the baseline value of $16,797 to approximately $21,000 (an increase of almost 25%). Similarly, from a societal perspective, the Bangladeshi minimum rural wage rate ($78.48 per month) would have to decrease by approximately 80% for the ICER (per live saved) to decrease to zero savings, see the Additional file 1 exhibit H.

## Discussion

This study compared the crèche intervention to the status-quo in cost-effectiveness analysis from both a program and societal perspective. Results showed that for a typical Bangladeshi population of 100,000 children aged 12 to 47 months who enrolled in crèches in rural areas, the intervention would cost $1.6 million annually ($16 per child) from the program perspective and would save 95.41 children from fatal drownings (equivalent to 2788.59 YLLs or DALYs averted). From the societal perspective, the intervention could generate approximately $15.9 million in cost savings to the community if parents freed up time was valued at the Bangladeshi minimum rural wage. This was equivalent to $166.7 thousand cost savings per live saved. From the program perspective alone, the crèches’ ICER per DALY averted, $575, is very cost-effective relative to a reference point of GDP per capita, which was $1248 in Bangladesh (World Health Organization 2015). Similarly, from the societal perspective excluding savings from parents freed up time, the crèches’ ICER per DALY averted, $1953, is cost-effective.

The parameter inputs that most impacted results were the opportunity cost of volunteers’ time (parents, community sensitization members, and crèche caregivers), the number of children per crèche, the number of lives saved, program non-administrative wages, and program maintenance costs. Overall, parents freed up time was one of the most important and sensitive factors. It was important because the economic savings produced for each user of the crèches were significantly greater than the cost per user. Similarly, while the social cost to community members and daycare caregivers was also important, this cost was lower compared to the economic savings generated by parents freed up time. In fact, even with the program's average attendance level of 58% of exposed children the cost savings were significant. Parents freed up time was also a sensitive parameter because it depended on the level of daycare attendance and the value of their free time. The attendance level could vary for different reasons, such as parent satisfaction with the quality of creche service, the level of sensitization activities in the community, etc. The value of parental free time could vary due to individual and contextual factors such as cost of living, economic opportunities available at each study site, religion, season, etc.

The results were also sensitive to the number of lives saved because there is a linear relationship between ICER and the number of survivors. The number of lives saved could vary if  the intervention is implemented in settings without the research component due to changes in the implementation package or in the intensity of monitoring, but this is an inherent problem in all evaluations. The effect of the intervention could also vary if other unobserved bias were not considered in the estimation of the effect size; however, we discussed why this is unlikely and our univariate sensitivity analysis showed that the difference in effect size would have to be significantly large (i.e., -84%) for the intervention to exceed the cost-effectiveness threshold. Finally, non-administrative wages (i.e., supervisors and monitoring officers) were the most influential program cost probably because the quality and ongoing operation of each crèche depended on the frequent monitoring and support provided by these staff positions.

These results are in line with a prior CEA in Bangladesh which compared a package of drowning prevention interventions, including the crèches, with the status-quo (Rahman et al. 2009). Both studies found a similar effect size for reducing fatal drownings (88% vs. 89%) but the prior study’s effect was attributed to the crèche and swimming lesson interventions implemented *together*, making the comparison of impact between both crèche interventions inadequate (Rahman et al. 2009; Alonge et al. 2020). Further, while both studies showed that about 80% of the program cost were wages, the cost per child estimate for that study was slightly higher than in this study ($51 in 2010 International USD, or $20–24 in 2015 USD, vs. $16). While both unit cost estimates produce cost-effective ICERs, the cost differences may be attributed to the costing methodology or amount of resources used to provide maintenance and support to the crèches.

The results of this study can be generalized to other crèche programs in rural Bangladesh using the same intervention inputs. Similarly, the results can be generalized to crèches in other countries if the economic conditions and cost of living are similar to those in rural Bangladesh. The univariate sensitivity analysis in this study may also guide future interventions in other settings on how the ICER may vary with changes in program costs or social perspective parameters.

Replicating the results of the crèches intervention in other settings would require incorporating the various components that contributed to its success. The crèche’s intervention success in reducing child drowning can be explained by several child protective effects, including provision of a safe environment away from water, supervision with capable child care, particularly during peak drowning hours, and community education about drowning risk expanded by active community engagement strategies (Hyder et al. 2014a; World Health Organization 2017; Saluja et al. 2004). In particular, the UIPCs and VIPCs played a major role in the sensitization of the community about both drowning prevention practices and dissemination of information about the crèches and their safety. The UIPCs also focus on building local support among community leaders and the VIPCs provided a standard and regular platform available to parents and community members to provide feedback about the crèche operations.

These CEA results are important for the fields of global health and injury prevention, especially for countries like Bangladesh with a high incidence of drowning. We make broad comparisons between our results and other cost-effectiveness analyzes, but care must be taken when comparing our results with individual studies due to the variety of methodological approaches, cost perspectives, cost inputs used, and differences in cost of living. Overall, our results indicate that from a societal perspective the crèche’s cost-effectiveness ratio per DALY averted (including economic savings from parents free up time), $-5703, is significantly more cost-effective compared to other injury prevention interventions for which cost-effectiveness ratios per DALY averted range between $5 to $556 (or $7 to $744 in 2015 $US) (Bishai and Hyder 2006). Excluding economic savings from parents freed up time, the crèche cost per DALY averted, $21,953, is higher than these child injury prevention interventions but also cost-effective. These other interventions include speed bumps, use of helmets, enforcement of traffic codes for road traffic injury prevention, and childproof containers for poisoning prevention (Bishai and Hyder 2006; Peden et al. 2008).

Compared to other child health interventions (e.g., treatment of febrile conditions, diarrheal disease, vaccines, severe acute malnutrition, platforms for delivery of interventions, etc.), with the cost per DALY averted estimates ranging between $8 for treatment of severe malaria up to $50,000 for sanitation improvement interventions, our CEA shows the crèches are cost-effective interventions to improve child health (Bishai and Hyder 2006; Zeng et al. 2018). To improve the comparison of cost and cost-effectiveness analysis results, more standardization of the methodologies used in future CEA is needed. Some of the resources that should be used to achieve this is following the CHEERS guidelines for CEA studies (Husereau et al. 2013). Further, the methods in this CEA study could guide other community-based injury prevention studies on how to conduct a CEA.

The lack of evidence-based intervention for drowning prevention (compared to other causes of child deaths) may have contributed to the lack of policy action to address childhood drowning in rural Bangladesh previously. The perception that interventions may not be readily targeted to address this issue, coupled with a limited global profile and funding for drowning prevention, may have further contributed to this lack of policy action until now. This study directly addresses some of these issues, and the cost-effectiveness results for the crèches in Bangladesh is likely to make a difference given the demand and renewed interest from different stakeholders, including the Bangladeshi government, non-governmental organizations, and global actors in Bangladesh, such as the World Health Organization in global drowning prevention (World Health Organization 2021; United Nations 2021). The effectiveness study contributed to effort by the Bangladeshi government (along with the Government of Ireland), Bloomberg Philanthropies and other key stakeholders to lead the successful effort at a new UN resolution on global drowning prevention, and efforts are currently underway to scale up the creche intervention in Bangladesh (Bloomberg 2021; World Health Organization 2021; United Nations 2021).

This CEA was limited by different factors. First, we used fatal drowning reduction estimates derived from a pre- and post-experimental design, which have inherent limitations. However, that study’s estimates accounted for the major sources’ bias and confounding, such as self-selected enrollment of children into crèches, secular trends (e.g., smaller family size or improved income), and unobserved factors associated with higher or lower drowning rates (e.g., local interventions or policies, etc.). Specifically, the estimation attempted reducing self-selection bias by controlling for the drowning rate of the crèche participant’s older sibling the year before the intervention. This control reduced the bias under the assumption that the reasons that parents chose to participate or not remained constant over time (Alonge et al. 2020). Further, recent studies from the same study sites showed that while all-cause under-five mortality decreased over the last 10 years that drowning trends remained generally the same (Rahman et al. 2019; Alonge et al. 2017b), suggesting secular trends did not impact drowning rates. Our sensitivity analysis showed that the intervention would remain very cost-effective if the number of drowning deaths averted fell by 52%, from 95 to 45 deaths, and cost-effective if it fell by 84%, from 95 to 25 deaths (see Additional file 1: Fig. SG2a-b). Lastly, the positive results are conservative given that the ICER excludes both improvement in YLDs and long-term socio-economic benefits. For example, crèches can offer protection against other child injuries (Rahman et al. 2012) and long-term economic benefits from improved childhood cognitive development and productivity in livelihood activities (Nair et al. 2017; Richter et al. 2017).

Further, there is a potential underestimation of the reduction in deaths from drowning with the crèches because the effectiveness estimates included all the respective deaths in the year among residents (i.e., it did not exclude deaths occurring on Fridays—the weekend day when crèches close). While the literature and the study data (not shown) suggested most deaths occured during crèche days (Saturday to Thursday), some also occurred on Friday. Hence, the number of lives saved would be higher if this fact was factored in. However, crèche effects on drowning are hypothesized to be both a direct effect of better supervision during crèche days and a spillover effect on non-crèche days as a result of some of the health education efforts tied to the creche. But, if the goal was to infer the direct effect of creches on deaths occurring on creche days only, then the estimation may be conservative.

This study also excluded healthcare costs associated with the medical treatment of drowning cases because likely there are few to no drowning survivors in rural Bangladesh. However, literature from high income countries where many more children survive near drowning, but suffer brain injury, suggests that the higher costs associated with drowning and other unintentional injuries are healthcare services and the lost productivity of survivors (Miller et al. 2000). Other indirect costs that cannot be monetized in our study may include reduced quality of life from pain, suffering, and social isolation.

Lastly, the study was limited by the data available to value parents freed up time. We approximated this value with the rural wage and varied this value in sensitivity analysis based on the distribution of 10 observations of local inflation adjusted GDPpc values. This assumption accounted for variation due to changes in the cost of living during recent years, but other factors (described above) are unaccounted for. The actual time allocation of parents freed up time in rural Bangladesh and estimates of its market value is unknown. Future studies evaluating what is the value of parents, especially mothers, freed up time are needed to better assess the cost savings with the intervention. Nonetheless, even when we excluded these cost savings from the results, the intervention remained cost-effective.

## Conclusion

This study showed the cost and cost-effectiveness of the large-scale implementation of the crèche intervention showing that crèches are cost-effective, even under scenarios of higher costs or lower effect sizes. Furthermore, the crèches have the potential to improve parental economic status by freeing up their childcare time. More research is necessary to determine the extent to which parents use this freed-up time in ways that benefit the household and the economy. Our present findings provide strong empirical support for investing in the scale up of crèches in communities burdened by high risk of child drowning.

## Supplementary Information

**Additional file 1.** Details about methods and results.

## Data Availability

The data used/or analyzed during the current study are available from the corresponding author on reasonable request and upon the approval of local implementation partners.

## Abbreviations

BDT: Bangladesh Taka
USD: United States dollars
LMICs: Low- and middle-income countries
SoLiD: Saving of Lives from childhood Drowning
UIPC: Union Injury Prevention Committees
VIPC: Village Injury Prevention Committees
GDP: Gross Domestic Product
RR: Risk ratio
WHO: World Health Organization
ICER: Incremental-cost-effectiveness ratio
CEA: Cost-effectiveness analysis
SoLiD: Saving of Lives from Drowning
RR: Risk ratios
SA: Sensitivity analysis
YLL: Years-of-life-lost
DALY: Disability-adjusted-life-year
