# Supplementary material for: Cost-effectiveness analysis of a large-scale crèche intervention to prevent child drowning in rural Bangladesh
Source: Inj Epidemiol. 2021 Oct 29;8:61. doi: 10.1186/s40621-021-00351-9 (PMC8555188; doi:10.1186/s40621-021-00351-9)
Supplement: Supplementary file 1 — Additional file 1. Details about methods and results. [file 40621_2021_351_MOESM1_ESM.docx]

**APPENDIX**

**Cost-Effectiveness Analysis of a Large-Scale Crèche Intervention to Prevent Child Drowning in Rural Bangladesh**

Contents

[**METHODS** 1](#_Toc80530191)

[**Appendix Exhibit A. Detailed List of Creche Program Inputs** 2](#_Toc80530192)

[**Appendix Exhibit B1. Societal Cost Parameter Assumptions for Monte Carlo Simulation** 3](#_Toc80530193)

[**Appendix Exhibit B2. Parent Productivity Cost Estimate** 3](#_Toc80530194)

[**Appendix Exhibit B3. Probability Density Function of the Distribution of Values of Key Data Inputs** 4](#_Toc80530195)

[**RESULTS** 4](#_Toc80530196)

[**Appendix Exhibit C. 10-Year Model of Annualized Program Cost, by Year** 5](#_Toc80530197)

[**Appendix Exhibit D. Child Enrollment and Capacity by Month** 5](#_Toc80530198)

[**Appendix Exhibit E. Program Cost by Study Area** 6](#_Toc80530199)

[**Appendix Exhibit F1. Area 1 Monthly Expenditure Trend** 7](#_Toc80530200)

[**Appendix Exhibit F2. Area 2 Monthly Expenditure Trend** 7](#_Toc80530201)

[**SENSITIVITY ANALYSIS** 8](#_Toc80530202)

[**Appendix Exhibit G1a. Effect of Changes to the Societal Cost on the ICER** 8](#_Toc80530203)

[**Appendix Exhibit G1b. Effect of Changes to the Program Cost on the ICER** 8](#_Toc80530204)

[**Appendix Exhibit G2a. Effect of Changes to the Number of DALYs Averted on the ICER** 9](#_Toc80530205)

[**Appendix Exhibit G2b. Effect of Changes to the Number of Drowning Deaths Averted on the ICER** 9](#_Toc80530206)

[**Appendix Exhibit H. Effect of Percentage Change of Inputs on the ICER** 10](#_Toc80530207)

[**CONSOLIDATED HEALTH ECONOMIC EVALUATION REPORTING STANDARDS – CHEERS CHECKLIST** 11](#_Toc80530208)

# **METHODS**

Program ingredients included experts’ time designing the intervention and developing the creche manuals, equipment for central offices (CO) and field offices (FOs), trainings for crèche mothers and assistants, supervisors and monitoring officers, expenses for coordinating union, upazilla and village injury committee meetings, engaging the community in the program, manual printing (one per crèche), and logistic supplies for the creches (tiffin box, plastic jug, mug, paper basket, bowl, soap case, belcha, plastic pack, melamine glass, story book, poster chart, floor mat (12x 16 ft2), black board, electric fan, board, switch, cable and normal bulb). Other items included in the program cost included supplies provided more than once per year (e.g. registration book, towels, toys, door mat, soap, broom, colors and paper, chalks), stipend for crèche mothers and assistant, CO and FOs’ rent, wages, utilities, maintenance.

## **Appendix Exhibit A. Detailed List of Creche Program Inputs**

| **Input Category** | | **Input Description** |
| --- | --- | --- |
| **Fixed Costs [life-time yrs.]** | | |
|  | Start-up | Program design, manual development, initial stakeholder meetings (2-6 months of labor and operational costs) [10] |
|  | Equipment | Office furniture [10] |
|  |  | Office computers [5] |
|  | Trainings | Crèche mother, mother assistant, trainer-of-trainers [5] |
|  | Crèche maintenance | Long lasting supplies: stationery, teaching and cleaning, equipment [5] |
|  |  | Short lasting supplies: teaching and cleaning supplies [1] |
|  |  | Repairs [2] |
| **Variable Cost (VC)** | |  |
|  | Wages / stipend (field staff) | Crèche mother & assistant |
|  |  | Supervisors |
|  |  | Monitoring officers |
|  | Community engagement | Upazila committee advocacy meeting (CAM), Union CAM, village CAM |
|  |  | Village and Union injury Prevention Committees (VIPC, UIPC) |
|  | Transportation | Excludes trainings |
|  | Field overhead | Rent, utilities, maintenance |
|  | Administration | Rent |
|  |  | Wages: Upazila coordinators, team leaders, senior FAO, A/C officer |
|  |  | Wages: Deputy team leaders, office assistants |
|  |  | Wages: Human Resources and Admin |
|  |  | Wages: Communication officer |
|  |  | Wages: Accountants |
|  |  | Utilities expenditure |
|  |  | Maintenance expenditure |
|  |  | Communication expenditure |

## **Appendix Exhibit B1. Societal Cost Parameter Assumptions for Monte Carlo Simulation**

Note: Negative values indicate savings. S.D. is the standard deviation. The three values highlighted in red are the values varied in sensitivity analysis. The 1^st^ two values were model using a probability density function. The last value, parent productivity, is a function of the first value in red (wages) and the number of children per crèche.

## **Appendix Exhibit B2. Parent Productivity Cost Estimate**

*The descriptive statistics (rows A, B, C, and D) for the parent opportunity cost are a function of the total number of children per crèche (2nd column), the percent of those children with each attendance level (5th column), and the sum of the payoff of each attendance level (7th column). The data across the rows is the same, except for the number of children per crèche (2^nd^ column). The number of children across rows A,B,C and D are based on the distribution of the monthly number of children per creche obtained from program records (the same data used in Table 2a in the manuscript. ** Data source: Alonge et al (2020). *** The payoff for "5+days" is the amount estimated in exhibit B1 (the minimum wage for part-time), for "2-4 days" (which is half the days) is half the part-time min. wage, and for "1- day" is the min. wage divided by 6 days to equal 1 day worth of part-time min. wage. The payoff values and the mean number of children in row A are the only numbers that varied in the Monte Carlos simulation.

## **Appendix Exhibit B3. Probability Density Function of the Distribution of Values of Key Data Inputs**

The Monte Carlos simulation truncated the distribution of values using the minimum and maximum values observed in the data of each input.

# **RESULTS**

Exhibits C shows the average monthly cost by input for the first three years of the program data and then the assumptions about the remaining 7 years in the 10-year projection model. We assumed that the annual program capacity for this annual expenditure level was the average of the mid. to full capacity reached during the 2^nd^ year for study area 2 and both the 2^nd^ and 3^rd^ year for area 1. The total annual cost was divided by the total annual capacity to produce the average cost.

## **Appendix Exhibit C. 10-Year Model of Annualized Program Cost, by Year**

## **Appendix Exhibit D. Child Enrollment and Capacity by Month**

## **Appendix Exhibit E. Program Cost by Study Area**

## **Appendix Exhibit F1. Area 1 Monthly Expenditure Trend**

## **Appendix Exhibit F2. Area 2 Monthly Expenditure Trend**

# **SENSITIVITY ANALYSIS**

## **Appendix Exhibit G1a. Effect of Changes to the Societal Cost on the ICER**

DALYs: Disability-adjusted-life years. YLL: Years-of-Life Lost; ICER: Incremental-Cost-Effectiveness-Ratio.

## **Appendix Exhibit G1b. Effect of Changes to the Program Cost on the ICER**

DALYs: Disability-adjusted-life years. YLL: Years-of-Life Lost; ICER: Incremental-Cost-Effectiveness-Ratio.

## **Appendix Exhibit G2a. Effect of Changes to the Number of DALYs Averted on the ICER**

DALYs: Disability-adjusted-life years. YLL: Years-of-Life Lost; ICER: Incremental-Cost-Effectiveness-Ratio. This figure shows that the intervention would remain very cost-effective if the number of DALYs (or drowning deaths) averted fell by less than 52%, from 2,789 to 1,327 DALYS, or from 95 to 45 deaths (the intersection between the green and red lines), and cost-effective if it fell by less than 84%, from 2,789 to 450 or from 95 to 25 deaths (the intersection between the red and blue lines). See Appendix Figures G2b to see the reduction in deaths for each percent reduction in the effect size.

## **Appendix Exhibit G2b. Effect of Changes to the Number of Drowning Deaths Averted on the ICER**

DALYs: Disability-adjusted-life years. YLL: Years-of-Life Lost; ICER: Incremental-Cost-Effectiveness-Ratio.

## **Appendix Exhibit H. Effect of Percentage Change of Inputs on the ICER**

# **CONSOLIDATED HEALTH ECONOMIC EVALUATION REPORTING STANDARDS – CHEERS CHECKLIST**
